# Modifiable risk factors for inpatient violence in psychiatric hospital: prospective study and prediction model

**DOI:** 10.1017/S0033291721002063

**Published:** 2023-01

**Authors:** Seena Fazel, Mark Toynbee, Howard Ryland, Maria Vazquez-Montes, Hasanen Al-Taiar, Achim Wolf, Omar Aziz, Vivek Khosla, Gautam Gulati, Thomas Fanshawe

**Affiliations:** 1 University of Oxford, University Dept, Warneford Hospital, Oxford, OX3 7JX, UK; 2 Oxford Health NHS Foundation Trust, Oxford, UK

**Keywords:** Inpatients, prediction, psychosis, risk assessment, violence

## Abstract

**Background:**

Violence perpetrated by psychiatric inpatients is associated with modifiable factors. Current structured approaches to assess inpatient violence risk lack predictive validity and linkage to interventions.

**Methods:**

Adult psychiatric inpatients on forensic and general wards in three psychiatric hospitals were recruited and followed up prospectively for 6 months. Information on modifiable (dynamic) risk factors were collected every 1–4 weeks, and baseline background factors. Data were transferred to a web-based monitoring system (FOxWeb) to calculate a total dynamic risk score. Outcomes were extracted from an incident-reporting system recording aggression and interpersonal violence. The association between total dynamic score and violent incidents was assessed by multilevel logistic regression and compared with dynamic score excluded.

**Results:**

We recruited 89 patients and conducted 624 separate assessments (median 5/patient). Mean age was 39 (s.d. 12.5) years with 20% (*n* = 18) female. Common diagnoses were schizophrenia-spectrum disorders (70%, *n* = 62) and personality disorders (20%, *n* = 18). There were 93 violent incidents. Factors contributing to violence risk were a total dynamic score of ⩾1 (OR 3.39, 95% CI 1.25–9.20), 10-year increase in age (OR 0.67, 0.47–0.96), and female sex (OR 2.78, 1.04–7.40). Non-significant associations with schizophrenia-spectrum disorder were found (OR 0.50, 0.20–1.21). In a fixed-effect model using all covariates, AUC was 0.77 (0.72–0.82) and 0.75 (0.70–0.80) when the dynamic score was excluded.

**Conclusions:**

In predicting violence risk in individuals with psychiatric disorders, modifiable factors added little incremental value beyond static ones in a psychiatric inpatient setting. Future work should make a clear distinction between risk factors that assist in prediction and those linked to needs.

## Introduction

Violence perpetrated by individuals with psychiatric disorders is common in inpatient settings. A review involving nearly 70 000 psychiatric inpatients from 122 studies found that the mean incidence of violence was 32%, with a rate of 183 events per 100 admissions per month (Bowers et al., 2011). Violence has significant adverse consequences on the victim, other patients, staff, and the institution as a whole, and can disrupt the care of the perpetrator (who may have to move to a more secure setting or custody) (Bowers et al., 2009). As part of any preventative strategy, improving assessment can help link those at the highest risk with additional treatments, and preserve resources by screening out those at low risk (Fazel et al., 2017), some of whom may be able to move to a less secure setting, including community-based ones (McDermott & Holoyda, 2014).

To complement and assist clinical decision-making, the use of structured tools and instruments have increasingly been advocated to improve risk assessment. Accordingly, they have been implemented in secure psychiatric settings, and in some more general adult inpatient units. A systematic review of these tools concluded that the predictive performance of those instruments predicting imminent violence, which relate to the next 24 h, such as the Brøset Violence Checklist (BVC) and the Dynamic Appraisal of Situational Aggression (DASA-IV), was better than more general and resource-intensive tools, such as HCR-20 and PCL-R, at separating out high- and low-risk patients (Ramesh, Igoumenou, Vazquez Montes, & Fazel, 2018). Certain national guidelines have recommended the use of imminent tools (National Institute for Health and Care Excellence, 2015). However, both BVC and DASA are based on one-off assessments of risk factors, use paper forms, and only predict risk over the very short-term (i.e. the next 24 h). Therefore they require very frequent administration over the course of an inpatient admission (Hvidhjelm, Sestoft, Skovgaard, & Bjorner, 2014; Nqwaku et al., 2018). This limits their usefulness, particularly the need for daily administration. Ultimately, the purpose of any risk assessment process is to enable clinicians to effectively target interventions that reduce the risks identified and to improve outcomes, rather than better prediction. Accurate predictions need to be assessed on this measure and require links to effective interventions. To date, randomised controlled trials in acute psychiatric inpatient settings provide contrasting findings − implementing standardised risk assessments can reduce subsequent violence in the Netherlands (Abderhalden et al., 2008) but not in Norway (Hvidhjelm et al., 2016). Both screening and structured professional judgement (SPJ) tools can potentially facilitate this process. SPJs typically include formulation and risk management, and provide a framework with which to address needs, while screening tools have the advantage that they are faster to administer and can be optimised to focus on predication rather than needs. This is particularly useful when frequent, repeated assessment of dynamic risk is required or to screen large numbers of individuals to inform resource allocation and improve the consistency and transparency of risk decisions.

To aid this, technological advancements have created opportunities for novel approaches to risk assessment (Danielsen, Fenger, Østergaard, Nielbo, & Mors, 2019; Wolf et al., 2018). Among these are the increasing capacity to monitor psychiatric symptoms and associated outcomes electronically (Fernandes et al., 2018; Miklowitz et al., 2012), incorporate automated alerting systems into electronic record systems, and for easier and clearer displays of longitudinal symptoms (Wang et al., 2020). These advances lend themselves to monitoring variation in symptoms and risk factors. In terms of risk assessment for key adverse outcomes, this allows for the possibility of inclusion of dynamic factors into tools. As they are potentially modifiable by clinical intervention, monitoring their variation may provide a focus for reducing risks and addressing clinical and psychosocial needs (Cullen et al., 2012).

In this paper, we describe the development and assessment of a simple, scalable risk monitoring tool for assessing violence risk in psychiatric inpatients in forensic and general adult wards and assess the incremental value of dynamic risk factors in this tool's predictive performance.

## Methods

### Developing a risk monitoring tool

Modifiable or dynamic risk factors with the strongest association with risk of violence were identified from a systematic review of violence in psychosis involving 110 studies and 45 533 individuals (Witt, van Dorn, & Fazel, 2013). These factors were used to formulate 10 questions to assess dynamic risk (see online Supplementary appendix B). These were all scored on a 5-point Likert scale between 0 and 4. Response options varied depending on the question. The timeframe for assessment was the period since the last such assessment. Some questions enquired about behaviour within this time period on an absolute scale, while others measured changes in specific behaviours since the previous assessment. For questions that asked if there was a change in a specific behaviour since the last review, no change or a decrease in the behaviour was scored as 0. To maximise scalability, the questions were designed to be answered by any mental health professional, such as a nurse, psychologist, medical doctor or occupational therapist, familiar with the patient, based on routinely collected information, without requiring an additional clinical interview.

The 10 questions on dynamic risk were then integrated into an online monitoring platform, FOxWeb (Forensic Oxford Web tool). A total dynamic score was calculated as the sum of the unweighted individual item scores. This platform had previously been shown to be easy-to-use and acceptable to clinicians in a pilot study of forensic psychiatric outpatients (Gulati et al., 2016). It provided a web-based interface for data collection and real-time graphical output in terms of bubblegrams (see online Supplementary Figs A1 and A2).

### Participants

The study participants consisted of adult (18 years old and older) inpatients on forensic and general adult wards within one UK National Health Service organisation (Oxford Health NHS Trust) over three hospital sites.

Twelve psychiatric wards (8 forensic and 4 general adult) were approached to take part: 4 male medium secure units, 2 male low secure unit and 2 female low secure units, and 1 male psychiatric intensive care unit, 1 male acute admissions unit and 2 female general adult wards. All forensic medium and low secure wards in Oxford Health NHS Foundation Trust were included. The general adult wards were selected to include a mixture of participants within acute adult inpatient services in the Trust. All forensic wards (79 beds) and 2 adult wards (43 beds) agreed to participate. All patients on the participating wards were approached and invited to take part in the study, unless nursing staff believed that a patient was not suitable to be approached. Patients provided informed consent to participate.

### Data collection

Data on historical information was recorded at baseline for descriptive purposes, including previous conviction for interpersonal violence, history of receiving inpatient treatment under the Mental Health Act, substance use disorder, alcohol use disorder, high baseline anger, and a history of self-harm (Witt et al., 2013). Baseline anger was determined by asking a clinician who knew the patient well if they showed evidence of feeling angry a lot of the time or often behaved in an angry manner (Novaco, 1994). Information was also recorded on the four static risk factors: calendar age, sex, ward type (general or forensic), and ICD-10 psychiatric diagnosis (main categories).

Assessment of 10 dynamic risk factors were undertaken by a member of the patient's clinical team. These raters were trainee psychiatrists, based on the wards participating in the study. One of these psychiatrists also inputted data directly into the FOxWeb system. Other data were inputted in the FOxWeb system by two medically qualified research assistants (MT, OA). Information was aimed to be collected weekly for patients on general wards and every 4 weeks for patients on forensic units. These timeframes were chosen to correspond to the frequency of multidisciplinary meetings in each setting. This was intended to provide a clinically meaningful interval, within which change in dynamic risk factors might reasonably be expected to occur. Each patient was followed up until the end of the study, discharge, transfer to another ward or when they withdrew consent. Data collection took place between March 2015 and December 2016.

### Outcome data

Information on outcomes was collected from the local health system's incident reporting system. The incident reporting system, Datix (Datix, 2019), is widely used by UK National Health Service hospitals and includes a number of categories for recording incidents. For the purposes of this project, an outcome was defined as an incident categorised on the Datix system as ‘violence’ or ‘aggression’. Violence-related incidents recorded in Datix in 17 different categories were included in this study. A full list of these categories is available in online Supplementary appendix A. The Datix system allowed staff to indicate if they came to the view that an individual was the instigator of the incident. The reliability of such designation is unclear, as staff are often not present from the start of an incident to accurately assess who started it. Incidents were therefore included regardless of whether the patient was deemed to be the instigator. This approach is consistent with evidence in psychiatric samples where perpetration and victim status overlap considerably, and that both are risk factors for each other (Sariaslan, Lichtenstein, Larsson, & Fazel, 2016). Incidents were only included in the analysis if they occurred within one week of an assessment of dynamic risk on a general ward, or four weeks on forensic wards. The outcome was linked to the most recent assessment of dynamic risk within these respective time periods.

### Ethics

The project was approved by the Oxford Research Ethics Committee on 18/12/2015 (ref: 15/SC/0051). The authors assert that all procedures contributing to this work comply with the ethical standards of the relevant national and institutional committees on human experimentation and with the Helsinki Declaration of 1975, as revised in 2008.

### Statistical analysis

A multilevel logistic model using patient as a random effect was fitted to assess the association between total dynamic score and occurrence of violent incidents, adjusting for age, sex, diagnosis (categorised dichotomously as schizophrenia-spectrum disorders *v.* other diagnostic categories), and type of ward (general *v.* forensic). This random term component of the model is the propensity of an individual to be involved in violent incidents, over and above the risk that can be attributed to the included measured variables. It takes into account the higher risk of repeat violent events in the same individual. The random effect term cannot be estimated without refitting the model each time for new datasets.

Separate models were fitted with the dynamic score treated as continuous; binary by dichotomising as > 0 *v.* 0; and binary by dichotomising as > 4 *v.* < = 4. A score of > 4 was chosen as this would indicate a change in at least two individual dynamic risk factors, as the maximum score for each factor is 4. The model with lowest deviance and Akaike information criterion (AIC) was selected. The results are presented as odds ratios (ORs) and 95% confidence intervals (CIs). Similar models were also fitted to assess the effect of each of the 10 continuously scored individual components of the dynamic score, adjusting for the same factors.

The predictive performance of the final model was compared against a similar model with the dynamic score excluded, to assess the effect of including the dynamic score. Predictive probabilities were obtained in two ways: firstly, incorporating the random and fixed effect terms to obtain a within-person predictive probability, and secondly by using the fixed effects only, to better indicate likely out-of-sample performance. Calibration plots comparing predicted and observed probabilities within deciles were constructed. The area under the receiver operating characteristic curve (ROC AUC), and its 95% CI, was also calculated.

Three different interactions were tested (sex and type of ward; sex and diagnosis; diagnosis and type of ward) but otherwise no variable selection was performed. The main analysis was repeated replacing total dynamic score with each of the 10 individual dynamic score items.

In addition, a survival analysis was performed, using a Cox proportional hazard model, to explore the time from last assessment to an incident occurring before next assessment, adjusted for repeated measures. This accounted for time at risk, as the follow-up periods were not similar for all patients. Hazard ratios, *p* values and 95% CIs were reported. The association between dynamic scores and an outcome defined as the patient being the instigator of the incident was also assessed by fitting multilevel logistic models similar to those described above.

We prepared a study protocol before the project started (see online Supplementary appendix C), and an analytic plan before any statistical analysis of the data (see online Supplementary appendix D).

## Results

The study cohort included 89 participants. Participants were mainly young (mean age 38.9 years [s.d. 12.5], median age 38 years [IQR 28, 51], range 19–62), 20% (*n* = 18) were female and the majority were on a forensic ward (75%, *n* = 67). Over two-thirds (70%, *n* = 62) had a primary diagnosis of a schizophrenia-spectrum disorder, with one in five (20%, *n* = 18) having a primary diagnosis of personality disorder (see Table 1). There were 93 violent incidents within one week (general wards) or four weeks (forensic wards) of an assessment (70%, *n* = 65 by females), perpetrated by 29 individuals (33%, 13 female and 16 male). Roughly half of the patients involved in violent incidents (14/29) had schizophrenia-spectrum disorder. The median number of incidents per patient was 2 (range 0–14) (see Table 1 and online Supplementary Table A1).

**Table 1.** Cohort demographic, clinical and background characteristics of 89 psychiatric inpatients

Age in years, mean (s.d.)	38.9 (12.5)
**Sex, *n* (%)**	
Male	71 (80%)
Female	18 (20%)
**Ward type, *n* (%)**	
Forensic	67 (75%)
General	22 (25%)
**Primary diagnosis, *n* (%)**	
Schizophrenia-spectrum disorder	62 (70%)
Substance use disorder	2 (2%)
Mood disorder	5 (6%)
Anxiety disorder	1 (1%)
Personality disorder	18 (20%)
Developmental disorder	1 (1%)
**Historical risk factors, *n* [*n* unknown] (%)**^a^	
Conviction for interpersonal violence	69 [1] (78%)
Previous treatment under the Mental Health Act	82 [1] (93%)
Substance use disorder	65 [2] (75%)
Alcohol use disorder	52 [4] (61%)
High baseline anger	54 [2] (62%)
History of self-harm	40 [4] (47%)

a Percentages calculated excluding individuals with missing data; [number of missing values].

We completed 624 separate assessments of dynamic risk factors. All 89 participants had at least one dynamic risk assessment, and the median number of assessments per patient was 5 (IQR 3–10). The distribution of total dynamic scores was highly positively skewed and ranged from 0 to 23 out of a maximum of 40 (median 2; IQR 0–6), with the most commonly scored individual items being anxiety, verbal or physical aggression, and impulsivity (see Table 2 and online Supplementary Table A2). Median follow up was 24 weeks (IQR 5–42) with a range of 0–81 weeks.

**Table 2.** Summary of dynamic scores for the 624 assessments of the study cohort

	Mean (s.d.)	Median (IQR)	N (%) scores > 0
1. Non-adherence with therapy	0.6 (1.1)	0 (0–1)	169 (27%)
2. Non-adherence with medication	0.3 (0.7)	0 (0–0)	89 (14%)
3. Aggression (verbal or physical)	0.7 (1.0)	0 (0–1)	244 (39%)
4. Discharge of tension and emotions	0.7 (1.1)	0 (0–1)	224 (36%)
5. Emergence/deterioration of paranoid/persecutory delusions	0.3 (0.6)	0 (0–0)	118 (19%)
6. Emergence/deterioration of hallucinations	0.2 (0.5)	0 (0–0)	73 (12%)
7. Increasing anger due to psychotic symptoms	0.2 (0.6)	0 (0–0)	81 (13%)
8. Emergence/increase in drug misuse	0.1 (0.4)	0 (0–0)	21 (3%)
9. Emergence/increase in alcohol misuse	0.0 (0.3)	0 (0–0)	14 (2%)
10. Increase in anxiety	1.0 (1.1)	1 (0–2)	354 (57%)
Overall dynamic total score (*n* = 624)	3.9 (4.7)	2 (0–6)	454 (73%)
Median dynamic total score per patient (*n* = 89)	2.6 (2.9)	2 (0–4)	

The relationship between total dynamic score and probability of violent incident occurrence appeared non-linear, and the best-fitting multilevel logistic model included total dynamic score as a binary variable (>0 *v.* 0). A non-zero dynamic score was associated with a higher probability of violent incidents (OR 3.39, 95% CI 1.25–9.20), and this was unaffected by adding any of the three tested interaction terms to the model. None of the interactions were significant. Female sex (OR 2.78, 95% CI 1.06–8.15) and lower age (OR 0.67 per 10 years increase, 95% CI 0.47–0.96) were associated with increased risk of violent incidents. None of the dynamic items was predictive on its own, either independently or when adjusted for other predictors (see Table 3).

**Table 3.** Associations between risk factors and occurrence of violent incidents

Variable	Adjusted odds ratio	95% confidence interval	*p* value
**Main model**			
Total dynamic score > 0 (v. 0 score)	3.39	1.25– 9.20	0.016
Age, per increase in 10 years	0.67	0.47–0.96	0.031
Female sex	2.78	1.04–7.40	0.041
Forensic ward (v. general ward)	0.71	0.23–2.21	0.560
Schizophrenia-spectrum disorder (v. other diagnoses)	0.50	0.20 to 1.21	0.125
Intercept	0.20	0.03–1.16	0.073
**Models with individual dynamic scores (per 1-point increase in item score)**			
Therapy non-adherence	1.13	0.90–1.42	0.291
Medication non-adherence	1.25	0.88–1.77	0.211
Aggression	1.26	0.97–1.65	0.083
Impulsivity	1.09	0.84–1.41	0.520
Paranoid delusions	1.14	0.73–1.79	0.564
Hallucinations	1.06	0.63–1.77	0.834
Anger due to psychosis	0.86	0.54–1.38	0.539
Drug misuse	1.53	0.81–2.87	0.186
Alcohol misuse	0.63	0.14–2.90	0.554
Anxiety	0.88	0.62–1.25	0.463

*Note:* Main model (upper part of the table) is a single multilevel logistic model, showing effects adjusted for the other variables listed. Models with individual dynamic scores (lower part) are from 10 similar models, after replacing the total dynamic score variable with each continuously scored individual item of the dynamic score in turn. These effects are also adjusted for age, sex, type of ward, and diagnostic category.

In survival analysis with outcome as the time until violent incident, the adjusted hazard ratio corresponding to a total dynamic score of greater than 0 was 2.29 (95% CI 0.74–7.13) (see online Supplementary Table A3 and Fig. A3). Participants were instigators in around half of the eligible violent incidents (48/93, 52%), but there was no evidence that higher dynamic scores were associated with being an instigator (see online Supplementary Table A4).

### Model performance and calibration

**Fig. 1.**
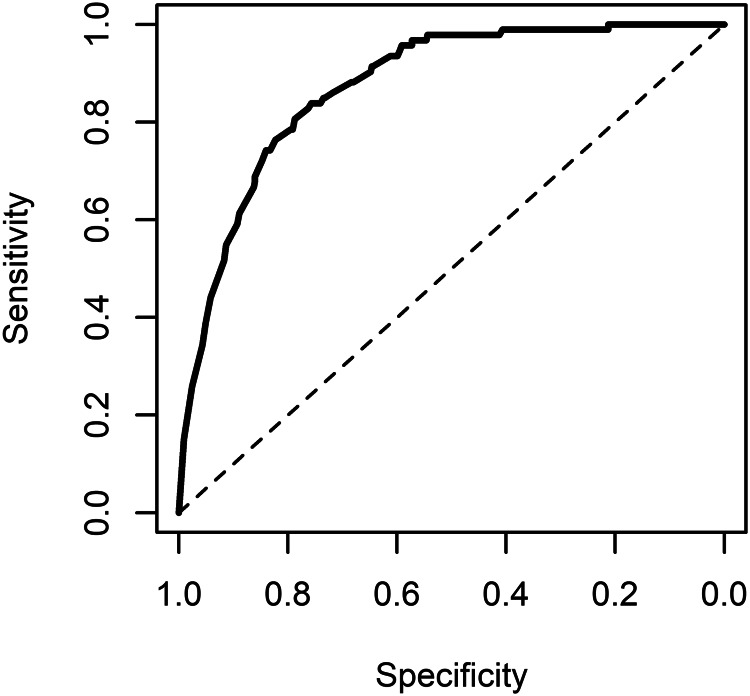
ROC curve of the main model predicting inpatient violence: predictions incorporating random effects. *Note*: AUC 0.87 (95% CI 0.84–0.91).

**Fig. 2.**
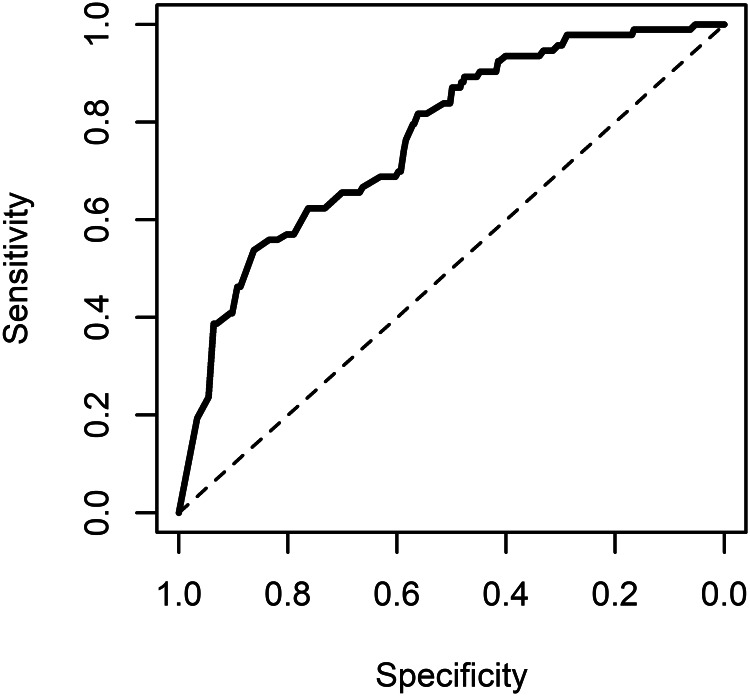
ROC curve of the main model predicting inpatient violence: predictions not incorporating random effects. *Note:* AUC 0.77 (95% CI 0.72–0.91).

The AUC of the main model for occurrence of violent incidents was 0.87 (95% CI 0.84–0.91) when incorporating the random effect term into the prediction (see Fig. 1). It was 0.77 (0.72–0.82) when using the fixed effect terms only (see Fig. 2), and 0.75 (0.70–0.80) using the fixed effect terms from the model without the dynamic score. The AUC values for the main model were almost identical to those obtained from models that treated the dynamic score as continuous or binary with a  > 4 threshold. Calibration was acceptable (see online Supplementary Fig. A4). PPVs (ranging from 0.31 to 0.44) and NPVs (ranging from 0.96 to 0.99) are presented in online Supplementary Table A2.

## Discussion

In this prospective study of 89 psychiatric inpatients followed up for 6 months, we conducted 624 assessments incorporating a range of static and modifiable risk factors for violence, and identified 93 incidents of subsequent inpatient violence. We developed and tested a prediction model that incorporated many modifiable or dynamic factors and examined its incremental performance over a model with four static factors. We also investigated whether simple changes to the prediction model could improve its accuracy.

We report two main findings. First, the tested dynamic factors, despite being based on the strongest available evidence, either as a total score or broken down into individual items, were not strongly predictive of violence risk. The binary score of the total dynamic score of more than 0 was predictive of increased risk, but not strongly enough to make a noticeable and practical difference to predictive probabilities. Second, the final prediction model's overall performance, which was good according to a range of metrics, was mainly driven by the static factors, in particular age and sex, and by other unmeasured person-specific factors represented by the model's random effect term. This was additionally shown when testing the discrimination of the model with only the measured risk factors. With the binary dynamic score, the model's performance shrunk very slightly from an AUC of 0.77 with this score to 0.75 without it. Female sex was associated with an increased risk of violent incidents, which is very different to community settings where such incidents are rare. Possible explanations for this include the possibility that women admitted to psychiatric hospitals have higher baseline risks and are more psychiatrically unwell than men. This is consistent with the finding that the total dynamic score was associated with higher risk for violent incidents.

There has been recent interest in using modifiable risk factors for managing risk of violence. However, this study does not support the view that modifying dynamic risk factors, with the approach taken of using continuous scores, would meaningfully assist in violence prediction. Nevertheless, monitoring dynamic factors may have other advantages apart from prediction, including needs assessments and formulation of management plans (Wong & Gordon, 2006). The lack of predictive power of these risk factors may result from their relative insensitivity to change, as indicted by the high proportion of participants scoring 0 on the total dynamic scale. In addition, clinical teams will typically act on these modifiable factors to prevent escalation, which will further dampen their predictive power. In predictive terms, the best-fitting model suggested that a score of 10 corresponded to no higher risk than a score of 1, suggesting it was too difficult to receive a high score for the measures to be sensitive enough. One approach to improve this would be to include and test other dynamic risk factors that are more sensitive to change, which could enhance the contribution of the dynamic score to risk prediction. Further work could explore factors that may show greater fluctuation within a relevant timeframe, such as interactions with staff and other patients (Papadopoulos et al., 2012), adherence to ward leave and other rules, and other markers of treatment engagement (Dack, Ross, Papadopoulos, Stewart, & Bowers, 2013). Another approach would be to use more sensitive instruments to measure the dynamic factors examined in this study, but at the cost of needing to conduct additional assessment. In addition, one included factor, anger due to psychosis, was not associated with violent outcomes in multivariable models, which may be explained by its links with community violence but no evidence for its association in inpatient settings (Witt et al., 2013). We reported previous violence to provide baseline information on the sample but did not use this factor in our prediction models.

Most inpatient units in high-income countries are familiar with electronic data entry and have the required resources to implement a tool such as FOxWeb, which requires internet access. The interface remained the same from a previous pilot in outpatients where it was found to be user-friendly by clinicians with minimal training (Gulati et al., 2016). These results indicate FOxWeb and similar electronic tools to be acceptable and scalable in a clinical setting. However, any violence risk assessment needs further work to link it to effective interventions, and ultimately to evaluate outcomes in a trial setting. Furthermore, such tools should be used as adjuncts, to support clinical decision-making, as they will never be able to capture the full range of individual risk factors for any particular patient (Fazel et al., 2017). Importantly, these tools should be developed with the aim of freeing up clinical time to focus on treatment, rather than as substitutes for clinical judgement or replacing it (Topol, 2019). As part of the implementation of a tool, linking it with additional management needs to be considered. This is a separate piece of work that will involve reviewing the literature on effective interventions, discussing within clinical teams the feasibility of such interventions and how they could be linked to any predictive tool, possible barriers and harms, and whether the interventions need adapting for local use.

There are several limitations to the current study. The risk factors were identified from a large systematic review and meta-analysis that was based on outpatient and inpatient settings (Witt et al., 2013) rather than solely inpatients. In this review, a quarter of the patients were in forensic wards and just under a third in inpatient settings. However, risk factors did not significantly change when only inpatient samples were used or focusing on severe violence rather than the broader definition used in this study. The sample included in this study was also diagnostically heterogeneous, while the systematic review from which we selected the risk factors to be tested only included studies of individuals with psychosis. In the current sample, a minority had non-psychotic illnesses, such as personality disorders (20%). Thus, the risk factors used to build the tool, identified from the systematic review, may not have included factors specific to these other patient populations. Future work could consider testing whether there are additional risk factors that would retain independence in multivariable models. The diagnostic heterogeneity can be viewed as a strength as it reflects the real-world situations of psychiatric wards. Further, no difference in violence rates was identified between adult and forensic wards. One possible explanation for the poor predictive accuracy of the total dynamic score (and individual dynamic risk factors) could be that any factors included in a risk tool should be based on data from a primary study rather than systematic review where confounders are not adequately adjusted for (Cornaggia, Beghi, Pavone, & Barale, 2011). The current investigation was based in one region, and adult and forensic inpatient wards, and will need replication in other settings. Overall, forensic patients were relatively over-represented, although the sample size was similar to other validation studies in the area (Ramesh et al., 2018). In considering generalisability, the average length of stay in the participating forensic wards was 1220 days and we did not have information on length of stay for the other included wards, which were standard general adult wards. Other possible limitations include that the psychiatric wards that agreed to participate may have done so because they were more active in developing and implementing risk management plans, compared to wards that chose not to participate. Similarly, patients were only included if they consented, and this may represent a population at lower risk, as those with higher risk due to factors such as more severe symptoms, may have been less likely to consent (McDermott, Gerbasi, Quanbeck, & Scott, 2005). Not all patients on each ward consented, and it is possible that there was ‘contamination’ in their risk management. However, we think that this is unlikely to have had a significant impact, as after the initial contact with the researcher there was no further contact between the patient and anyone not part of their normal clinical team. The information to complete FOxWeb was obtained at intervals of one to four weeks and took a few minutes per patient. It is unlikely therefore that the management of either consented or non-consented patients was affected by the data collection. Another limitation is the use of a broad definition of violence, which included verbal aggression. This allows for increased accuracy of prediction and reflects the view that all forms of violence and aggression are disruptive and potentially harmful in an inpatient setting. The outcome data was based on routinely collected information, which will lead to underreporting. As outcome data from the Datix system relied on staff to report incidents, this will likely have resulted in an underestimate of the number of violent incidents. There may also have been variation between wards and individual staff members of the threshold for reporting an incident through the Datix system. In addition, it included victimisation data as it was often not possible to distinguish perpetration from victimisation because they overlapped. To check this, we conducted a sensitivity analysis looking at incidents that were solely coded as perpetration and found no material difference in the magnitude and direction of risk factors. Consistent with other studies of violent outcomes, we included verbal aggression and threats. We did not measure inter-rater reliability, although clinicians who knew the patients provided the tool responses to researchers who entered the data. The prediction model, with or without dynamic factors, requires further internal validation, using resampling techniques like bootstrapping or cross-validation, and adjustment for optimism.

In practice, any prediction model would be based on the measured risk factors with or without a binary dynamic factor score (greater than 0 *v.* 0). The static element of the model was simple and scalable, and included only four items – age, sex, type of ward, and main psychiatric diagnostic category. In contrast, the dynamic component was based on 10 questions on modifiable risk factors, which would require additional assessment. Although the static component may assist in providing baseline cross-sectional measures of risk, it does not assist in guiding management.

## Conclusion

We have developed a scalable approach to monitoring risk of violence in psychiatric inpatients (FOxWeb), which requires further testing in independent external samples. Although the risk assessment incorporated many dynamic risk factors, these did not provide incremental discriminative power for predicting violence beyond the information provided by a few static factors. Future work should make a clear distinction between factors that assist in prediction and those that are more closely linked to needs.

## Supplementary material

For supplementary material accompanying this paper visit https://doi.org/10.1017/S0033291721002063. click here to view supplementary material
