# Supplementary material for: Modifiable risk factors for inpatient violence in psychiatric hospital: prospective study and prediction model
Source: Psychol Med. 2021 May 24;53(2):590–6. doi: 10.1017/S0033291721002063 (PMC9899559; doi:10.1017/S0033291721002063)
Supplement: Supplementary file 1 [file S0033291721002063sup001.docx]

**Appendix A**

Additional Figures and Tables

**Figure A1:** FOxWeb data input screen

**
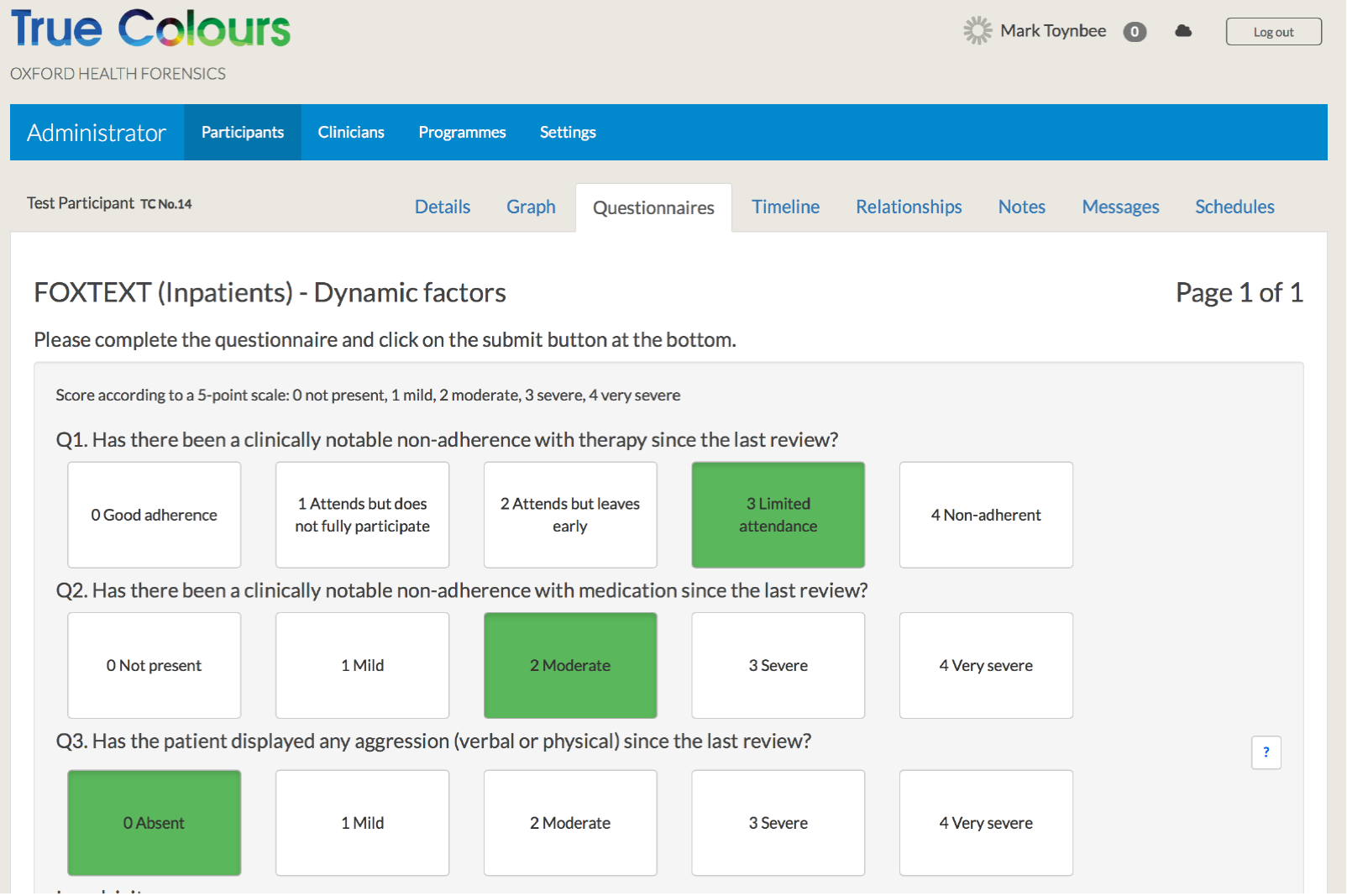
**

**Figure A2:** FOxWeb data monitoring summary for a simulated individual in the study cohort

**
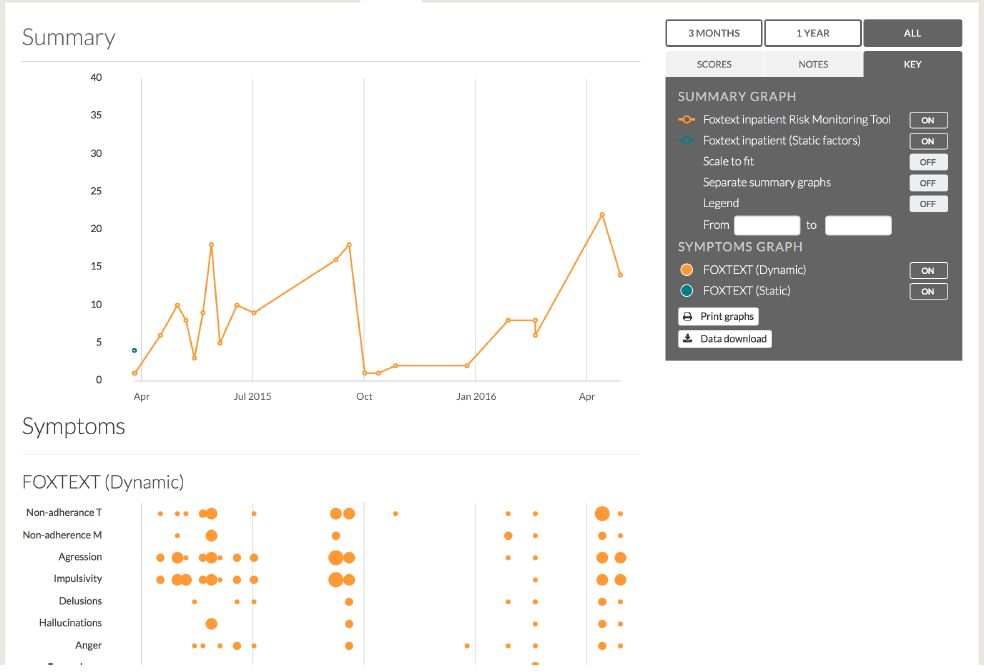
**

**Figure A3**: Kaplan-Meier plots showing association between dichotomous total dynamic score and violent outcomes (y axis)

|  |  |
| --- | --- |

Note: DynSc = total dynamic score on FOxWeb

**Figure A4**: Calibration plot of observed probabilities against predicted probabilities

| 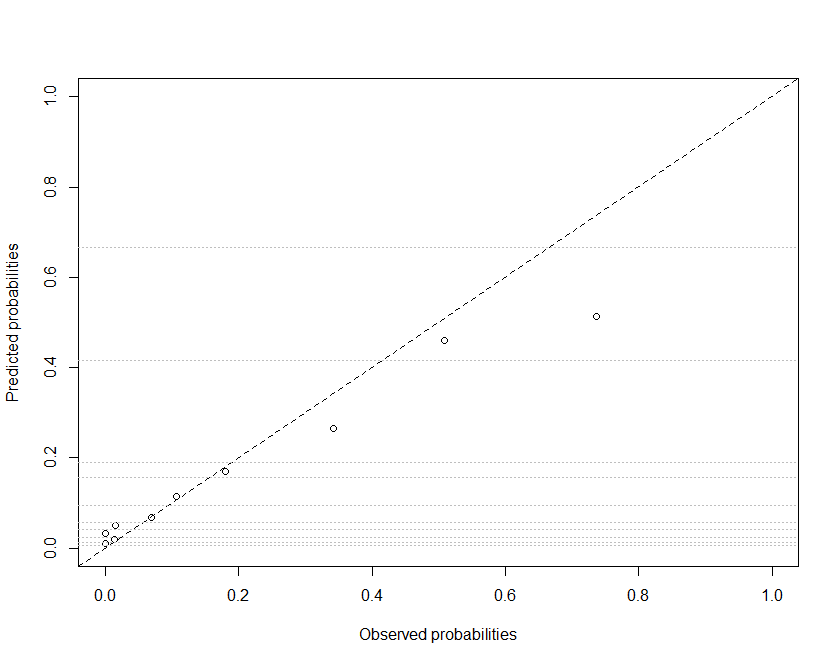 |
| --- |

## Note: Predicted probabilities from the multilevel logistic model were averaged within deciles of their overall distribution, ignoring the repeated measure structure. These averages were then plotted against the percentage of incidents within the corresponding deciles. Grey dotted lines represent deciles of predicted probabilities. The diagonal line represents perfect calibration.

**Table A1:** Datix codes and categories

| Datix code | Datix category |
| --- | --- |
| VA001 | Verbal Abuse Patient on Patient |
| VA002 | Verbal Abuse Patient on Staff |
| VA005 | Verbal Abuse Patient on Public |
| VA009 | Violence No Injury Patient on Staff |
| VA012 | Violence No Injury Patient on Patient |
| VA014 | Violence No Injury Patient on Public |
| VA015 | Violence With Injury Patient on Patient |
| VA016 | Violence With Injury Patient on Staff |
| VA018 | Violence With Injury Public on Patient |
| VA021 | Violence Patient Towards Property |
| VA023 | Verbal Abuse Racial Patient on Staff |
| VA024 | Violence No Injury Racial Patient on Staff |
| VA027 | Violence With Injury Racial Patient on Staff |
| VA028 | Alleged Assault on Patient |
| VA029 | Alleged Assault on Staff |
| VA031 | Disturbed Agitated Outburst Behaviour |
| VA036 | Threat of Violence |

**Table A2**: Characteristics of patients with at least one recorded incident

|  | **Total sample** | **Number (%) with at least one incident** | **Median (IQR) number of incidents per patient^1^** |
| --- | --- | --- | --- |
| **Overall** | 89 | 29 (33%) | 2 (1-4) |
| **Female** | 18 | 13 (72%) | 4 (2-7) |
| **Male** | 71 | 16 (23%) | 1 (1-2) |
| **Psychotic disorder** | 62 | 14 (23%) | 2 (1-2) |
| **Other diagnosis** | 27 | 15 (56%) | 3 (1-5) |
| **Forensic** | 67 | 24 (36%) | 2 (1-4.5) |
| **Acute** | 22 | 5 (23%) | 2 (1-3) |
| **Age, years** |  |  |  |
| **≤20** | 3 | 2 (67%) | 9 (8-10) |
| **>20, ≤30** | 26 | 11 (42%) | 2 (1-6) |
| **>30, ≤40** | 21 | 5 (24%) | 2 (1-3) |
| **>40, ≤50** | 14 | 6 (43%) | 2 (2-3) |
| **>50, ≤60** | 21 | 5 (24%) | 1 (1-2) |
| **60+0** | 4 | 0 (0%) | -- |

^1^ Calculated among patients with at least one recorded incident

**Table A3:** Summary of dynamic scores (total and for each item) for the whole sample and in patients with and without involvement in violent incidents

|  | **Overall**  **N=624 assessments** | | **Inpatients involved in one or more violent incidents**  **N=93 assessments** | | **Inpatients not involved in violent incidents**  **N=531 assessments** | |
| --- | --- | --- | --- | --- | --- | --- |
|  | **Median (IQR)** | **N (%) of scores >0** | **Median (IQR)** | **N (%) of scores >0** | **Median (IQR)** | **N (%)**  **of scores >0** |
| Total dynamic score | 2 (0-6) | 454 (73%) | 5 (2-11) | 87 (94%) | 2 (0-5) | 376 (69%) |
| *Individual dynamic score items* |  |  |  |  |  |  |
| Therapy non-adherence | 0 (0-1) | 169 (27%) | 0 (0-2) | 45 (48%) | 0 (0-0) | 124 (23%) |
| Medication non-adherence | 0 (0-0) | 89 (14%) | 0 (0-1) | 24 (26%) | 0 (0-0) | 65 (12%) |
| Aggression | 0 (0-1) | 244 (39%) | 1 (0-2) | 68 (73%) | 0 (0-1) | 176 (33%) |
| Impulsive emotional outburst | 0 (0-1) | 224 (36%) | 1 (0-2) | 58 (62%) | 0 (0-1) | 166 (31%) |
| Paranoid delusions | 0 (0-0) | 118 (19%) | 0 (0-0) | 21 (23%) | 0 (0-0) | 97 (18%) |
| Hallucinations | 0 (0-0) | 73 (12%) | 0 (0-0) | 11 (12%) | 0 (0-0) | 62 (12%) |
| Anger due to psychosis | 0 (0-0) | 81 (13%) | 0 (0-0) | 18 (19%) | 0 (0-0) | 63 (12%) |
| Substance misuse | 0 (0-0) | 21 (3%) | 0 (0-0) | 5 (5%) | 0 (0-0) | 16 (3%) |
| Alcohol misuse | 0 (0-0) | 14 (2%) | 0 (0-0) | 1 (1%) | 0 (0-0) | 13 (2%) |
| Anxiety | 0 (0-2) | 354 (57%) | 2 (1-3) | 70 (75%) | 1 (0-2) | 284 (53%) |

**Table A4**: Association between risk factors and violence outcomes

|  | **Adjusted hazard ratio** | **95% confidence interval** | **p-value** |
| --- | --- | --- | --- |
| **Total dynamic score > 0** | 2.29 | 0.74 to 7.13 | 0.152 |
| **Age, per 10 years** | 0.73 | 0.52 to 1.03 | 0.072 |
| **Female sex** | 3.40 | 1.45 to 7.98 | 0.005 |
| **Forensic ward** | 0.32 | 0.10 to 1.00 | 0.049 |
| **Psychotic disorder** | 0.42 | 0.19 to 0.93 | 0.031 |

Results obtained fitting a Cox Proportional Hazard model to multiple-record-per-subject survival data. Assessment date defined the starting of exposure time. Incident date defined the time of event occurrence, which was censored at the next assessment date if no incident was observed within the pre-specified interval of one week for acute and four weeks for forensic wards.

**Table A5**: Associations between being an instigator and total dynamic score (continuous and dichotomised), and individual dynamic score items. All effects are adjusted for age, sex, type of ward and diagnosis.

| **Variable** | **Odds ratio** | **95% confidence interval** | **p-value** |
| --- | --- | --- | --- |
| **Total dynamic score** | 0.99 | 0.93 to 1.06 | 0.794 |
| **Total dynamic score > 0** | 0.50 | 0.14 to 1.88 | 0.308 |
| **Total dynamic score > 4** | 0.83 | 0.42 to 1.64 | 0.587 |
| ***Individual dynamic score items*** |  |  |  |
| **Therapy non-adherence** | 0.95 | 0.74 to 1.23 | 0.707 |
| **Medication non-adherence** | 1.16 | 0.76 to 1.76 | 0.484 |
| **Aggression** | 0.78 | 0.89 to 1.04 | 0.094 |
| **Impulsive emotional outburst** | 0.99 | 0.75 to 1.31 | 0.953 |
| **Paranoid delusions** | 1.23 | 0.72 to 2.10 | 0.454 |
| **Hallucinations** | 1.50 | 0.85 to 2.67 | 0.164 |
| **Anger due to psychosis** | 1.09 | 0.68 to 1.75 | 0.712 |
| **Substance misuse** | 0.38 | 0.12 to 1.24 | 0.108 |
| **Alcohol misuse** | 0.69 | 0.22 to 2.16 | 0.524 |
| **Anxiety** | 0.99 | 0.68 to 1.43 | 0.940 |

**Appendix B**

FOxWeb Dynamic Factors – 10 Assessment Questions

| 1 | Has there been a clinically notable non-adherence with therapy since the last review? |
| --- | --- |
| 2 | Has there been a clinically notable non-adherence with medication since the last review? |
| 3 | Has the patient displayed any aggression (verbal or physical) since the last review? |
| 4 | Has the patient had any sudden, unmodulated, arbitrary, misdirected discharge of tension and emotions without concern about consequences since the last review? |
| 5 | Has there been either an emergence of, or deterioration in relation to, paranoid or persecutory delusions since the last review? |
| 6 | Has there been either an emergence of, or deterioration in relation to, hallucinations since the last review? |
| 7 | Is there any evidence of increasing anger due to psychotic symptoms since the last review? |
| 8 | Has there been an emergence/increase in drug misuse since the last review? |
| 9 | Has there been an emergence/increase in alcohol misuse since the last review? |
| 10 | Has there been a clinically notable increase in anxiety since the last review? |

Each question with response options:

1. Has there been a clinically notable non-adherence with therapy* since the last review?

| 0 | Good adherence |
| --- | --- |
| 1 | Attends but does not fully participate |
| 2 | Attends but leaves early |
| 3 | Limited attendance |
| 4 | Non-adherent |
| U | Not assessed |

*any non-adherence with the agreed treatment plan other than medication, such as ward round attendance or psychotherapy

2. Has there been a clinically notable non-adherence with medication since the last review?

| 0 | Not present |
| --- | --- |
| 1 | Mild |
| 2 | Moderate |
| 3 | Severe |
| 4 | Very severe |
| U | Not assessed |

3. Has the patient displayed any aggression (verbal or physical) since the last review?

| 0 | Not present |
| --- | --- |
| 1 | Mild |
| 2 | Moderate |
| 3 | Severe |
| 4 | Very severe |
| U | Not assessed |

4. Has the patient had any sudden, unmodulated, arbitrary, misdirected discharge of tension and emotions without concern about consequences since the last review?

| 0 | Absent or Minimal (questionable pathology; may be at the upper extreme of normal limits). |
| --- | --- |
| 1 | Mild – Patient tends to be easily angered and frustrated when facing stress or denied gratification but rarely acts on impulse. |
| 2 | Moderate – Patient gets angered and verbally abusive with minimal provocation. May be occasionally threatening, destructive, or have one or two episodes involving physical confrontation or a minor brawl. |
| 3 | Severe – Patient exhibits repeated impulsive episodes involving verbal abuse destruction of property, or physical threats. There may be one or two episodes involving serious assault, for which the patient requires isolation, physical restraint or p.r.n. sedation. |
| 4 | Very Severe – Patient frequently is impulsively aggressive, threatening, demanding, and destructive, without any apparent consideration of consequences. Shows assaultive behaviour and may also be sexually offensive and possibly respond behaviourally to hallucinatory commands. Or patient exhibits homicidal attacks, sexual assaults, repeated brutality, or self-destructive behaviour. Requires constant direct supervision or external constraints because of inability to control dangerous impulses. |
| U | Not assessed |

5. Has there been either an emergence of, or deterioration in relation to, paranoid or persecutory delusions since the last review?

| 0 | Absent |
| --- | --- |
| 1 | Mild |
| 2 | Moderate |
| 3 | Severe |
| 4 | Very severe |
| U | Not assessed |

6. Has there been either an emergence of, or deterioration in relation to hallucinations since the last review?

| 0 | Absent |
| --- | --- |
| 1 | Mild |
| 2 | Moderate |
| 3 | Severe |
| 4 | Very severe |
| U | Not assessed |

7. Is there any evidence of increasing anger due to psychotic symptoms since the last review?

| 0 | Absent |
| --- | --- |
| 1 | Mild |
| 2 | Moderate |
| 3 | Severe |
| 4 | Very severe |
| U | Not assessed |

8. Has there been an emergence/increase in drug misuse since the last review?

| 0 | Not present - indicated abstinence |
| --- | --- |
| 1 | Mild - occasional or minimal use that was not considered problematic by clinicians. |
| 2 | Moderate - persistent use despite clearly associated problems. |
| 3 | Severe - regular excessive consumption or binges, with severity associated problems, and indicated substance dependence. |
| 4 | Very severe - regular excessive consumption or binges, with severity associated problems, and indicated substance dependence. |
| U | Not assessed |

9. Has there been an emergence/increase in alcohol misuse since the last review?

| 0 | Not present |
| --- | --- |
| 1 | Mild |
| 2 | Moderate |
| 3 | Severe |
| 4 | Very severe |
| U | Not assessed |

10. Has there been a clinically notable increase in anxiety since the last review?

| 0 | Absent |
| --- | --- |
| 1 | Mild |
| 2 | Moderate |
| 3 | Severe |
| 4 | Very severe |
| U | Not assessed |

**Appendix C**

Study Protocol

**Study Title:**  Assessing risk of violence in forensic psychiatric patients (Foxweb)

**Internal Reference Number / Short title:** Foxweb

**Ethics Ref:**

**Date and Version No:** 09/12/14 version 1

| **Chief Investigator:** | Professor Seena Fazel  University of Oxford, Department of Psychiatry  Seena.fazel@psych.ox.ac.uk |
| --- | --- |
| **Investigators:** | Dr Omar Ali Aziz, Dr Vivek Ghosla, Dr Hasanen Al Tair, Dr Gautam Gulati |
| **Sponsor:** | University of Oxford/ |
| **Funder:** | Department of Psychiatry. No specific funding as this is part of Dr Aziz’s MSc. SF is funded by the Wellcome Trust. |
| **Chief Investigator Signature:** | Professor Seena Fazel |

**Confidentiality Statement**

This document contains confidential information that must not be disclosed to anyone other than the Sponsor, the Investigator Team, host organisation, and members of the Research Ethics Committee, unless authorised to do so.

**TABLE OF CONTENTS**

[1. SYNOPSIS 3](#_Toc405216765)

[2. BACKGROUND AND RATIONALE 3](#_Toc405216766)

[3. OBJECTIVES AND OUTCOME MEASURES 4](#_Toc405216767)

[4. STUDY DESIGN AND PROCEDURE 4](#_Toc405216768)

[5. PARTICIPANT IDENTIFICATION 5](#_Toc405216769)

[5.1. Study Participants 5](#_Toc405216770)

[5.2. Inclusion Criteria 5](#_Toc405216771)

[5.3. Exclusion Criteria 5](#_Toc405216772)

[6. STUDY PROCEDURES 5](#_Toc405216773)

[6.1. Recruitment and Informed Consent 7](#_Toc405216774)

[6.2. Discontinuation/Withdrawal of Participants from Study 8](#_Toc405216775)

[6.3. Definition of End of Study 8](#_Toc405216776)

[7. STATISTICS AND ANALYSIS 8](#_Toc405216777)

[7.1. Description of Statistical Methods 8](#_Toc405216778)

[8. DATA MANAGEMENT 9](#_Toc405216780)

[8.1. Access to Data 9](#_Toc405216781)

[8.2. Data Recording and Record Keeping 9](#_Toc405216782)

[9. ETHICAL AND REGULATORY CONSIDERATIONS 9](#_Toc405216783)

[9.1. Declaration of Helsinki 9](#_Toc405216784)

[9.2. Guidelines for Good Clinical Practice 9](#_Toc405216785)

[9.3. Approvals 9](#_Toc405216786)

[9.4. Reporting 9](#_Toc405216787)

[9.5. Participant Confidentiality 9](#_Toc405216788)

[10. FINANCE AND INSURANCE 10](#_Toc405216789)

[10.1. Funding 10](#_Toc405216790)

[10.2. Insurance 10](#_Toc405216791)

# SYNOPSIS

| **Study Title** | Assessing risk of violence in forensic psychiatric patients (Foxweb) | |
| --- | --- | --- |
| **Internal ref. no. / short title** |  | |
| **Study Design** | Qualitative research involving questionnaire | |
| **Study Participants** | 120 patients and 10-20 staff | |
| **Planned Sample Size** | 120 | |
| **Planned Study Period** | 6-12 months | |
|  | **Objectives** | **Outcome Measures** |
| **Primary** | To assess risk factors for inpatients violence in forensic psychiatric units | 10 item validated questionnaire on risk factors |
| **Secondary** | To see if changes in risk factors correlate with critical incidents | Critical incidents in inpatient settings |

# BACKGROUND AND RATIONALE

Most forensic psychiatric patients reside in secure hospitals (also called high, medium, and low secure units) where they are managed by multidisciplinary mental health teams. Such patients are usually admitted from three main areas: (a) court after a criminal offence, (b) other psychiatric units where they have been violent or at risk of violence, and (c) occasionally directly from prison. Such patients cost around four times other psychiatric patients, and part of the reason for this is the high rates of violent incidents to staff and other patients in such units. The current research project builds on work in the Department of Psychiatry Oxford that has a web-based tool (called OxText) to gather patient information as a means to improve patient self-management and symptom monitoring. For the purposes of this project, we intend to develop a system for clinical staff to collect information on certain symptoms (or risk factors) from inpatients using a web-based interface and an application that has already been developed by the OxText team for other projects, and by our team as part of a service evaluation project for outpatients in forensic psychiatry. In addition, information on critical incidents that are routinely collected by clinical staff (and uploaded onto a Trust-wide database) will be extracted and linked to the Foxweb. This would enable information on a particular individual's risk factors from Foxweb to be compared with that patient's subsequent violent and other serious incidents to see what risk factors are most predictive of violence in forensic psychiatric patients.

Foxweb was developed based on a systematic review of risk factors for inpatient violence that Professor Fazel was involved in and published last year (Witt, PLoS One 2013). From this review, all the strongest modifiable risk factors were extracted, and validated questions asking about these risk factors were then collated. Foxweb involves 10 dynamic factors scored on a rating scale of 0-5 using standardised questions from validated instruments/questionnaires, and 5 static factors (only completed once). A slightly amended version of this instrument also exists for outpatients, which also includes 10 dynamic factors and 5 static factors, and has been piloted in over 80 patients as part of a service evaluation project in 2014. The instruments have been developed as a web-based tool, and take between 2-5 minutes to complete.

The scientific justification for this research is to develop a reliable, feasible, accurate and scalable way of assessing and monitoring risk of violence in forensic psychiatric patients. This will enable staff involved with the care of these patients to identify a patient who is becoming likely to engage in violence and then take the appropriate means to intervene before an incident occurs. This will not only potentially improve the health of the patient being monitored, but other patients as well as staff who may be victims of any violence. It may also assist in saving money by saving clinical time by having easily accessible patient information on risk, and by possibly reducing critical incidents in inpatient units.

# OBJECTIVES AND OUTCOME MEASURES

| **Objectives** | **Outcome Measures** | **Timepoint(s) of evaluation of this outcome measure (if applicable)** |
| --- | --- | --- |
| To assess risk factors for inpatients violence in forensic psychiatric units | 10 item validated questionnaire on risk factors (that we are calling Foxweb) | 3-6 months |
| **Secondary Objective**  To see if changes in risk factors correlate with critical incidents | Critical incidents in inpatient settings  These are routinely collected by staff and uploaded into a Trust-based database called ‘Safeguard’. Critical incidents include interpersonal violence, damage to property, absconding, and self harm. |  |

# STUDY DESIGN

This project will be a questionnaire study, where a questionnaire is administered to the study participants in order to collect information about a certain group of patients. The questionnaire will involve items about a patients risk factors for violence. This data will then be correlated with routinely downloaded data on critical incidents involving those patients and then analysed. The study participants will be both forensic psychiatric patients as well as members of staff. The questionnaire will be administered to the members of staff, asking them questions about individual patients and their risk factors for violence.The study will take 4-6 months.

Utilising Foxweb (a web based instrument containing items on risk factors for violence), data will be collected regularly on inpatients in forensic psychiatric wards that are part of Oxford Health NHS Foundation Trust (Oxford Clinic, Thames House, Wenric House, Lambourne House, Woodlands House, and Malborough House). The instrument will not be directly administered to patients, but rather one of the researchers will ask a member of the clinical team about a particular patient. The questions are based on information that will have been already routinely collected by the clinical team. Thus, the staff member nor the researcher will interview the patient – rather, the staff member will simply answer questions about a particular patient based on information already collected.

Inpatients in the Trust will be followed up regularly using the same tool to assess change in risk factors for 6-12 months. The frequency of this has not been exactly determined but likely to be every 1-4 weeks, depending on the nature of the ward. In acute wards, weekly scores will be appropriate. In more longer stay wards, then this may be every 3-4 weeks. At the end of follow up, we will download routinely collected information on critical incidents (violence towards staff, other patients and property, self harm, and absconding) from a Trust-based database called 'Safeguard'. We will then examine associations between risk factors and critical incidents, particularly those involving violence towards others.

# PARTICIPANT IDENTIFICATION

## Participants

The study participants consist of patients who are inpatients in a forensic psychiatric unit and member of direct care team .The total number of patients will depend on how many agree to consent to the study, but it will be a maximum of 128 as this is the current number of forensic psychiatric inpatients in Oxford Health NHS Foundation Trust. Approximately 10-20 member of direct care team will be needed for this study.

## Inclusion Criteria

Patient Participants

-Participant is willing and able to give informed consent for participation in the study

-Male or female aged 18 years and above

-Inpatient in a forensic psychiatric ward

Staff Participants

-Participant is willing and able to give informed consent for participation in the study

-Male or female aged 18 years and above

-Member of care team on a forensic psychiatric ward

## Exclusion Criteria

Patient Participants

-Non-English speaking patients

Unfortunately patients who cannot speak English will not be able to consent and understand the patient information leaflet. Utilising interpreters will be difficult to arrange

Staff Participants

-Staff who are not involved with direct clinical care of a patient

# STUDY PROCEDURE

The only study procedures will be (See also Appendix A):

- Taking informed consent and organising to meet the direct member of the patients care team
- Using a web based tool, and going through a list of questions with the direct care team on the iPad. This process should only take a maximum of 2 minutes per patient
- Repeating this on a regular basis (1-4 weekly)

## Recruitment

**Patient participants:**

Patients on a forensic psychiatric unit will be approached individually by a member of their care team, who will ask the patient whether they will be fine with being approached by a researcher to speak about the study. If the patient agrees the researcher will approach them, introduce himself and explain the study as well as giving an information sheet as well as consent sheet. The patient will be given as much time as needed to decide participation in the study. Consent will be recorded in writing by the research clinicians.

**Staff participants:**

Member of direct care team will be recruited via the researcher going to the individual wards and presenting the proposed project to the ward team, consisting of the consultants, key nurses, SHO’s etc. The researcher will then hand out the staff information sheet, and who is interested can sign the consent form and be part of this research. This will be arranged through the ward managers who are already aware of this research and have agreed to help the project. Approximately 10-20 staff will be needed for this study.

## Informed Consent

Written and verbal versions of the Participant Information and Informed Consent will be presented to the participants detailing no less than: the exact nature of the study; what it will involve for the participant; the implications and constraints of the protocol; the known side effects and any risks involved in taking part. It will be clearly stated that the participant is free to withdraw from the study at any time for any reason without prejudice to future care, and with no obligation to give the reason for withdrawal.

The participant will be allowed as much time as wished to consider the information, and the opportunity to question the Investigator, their GP or other independent parties to decide whether they will participate in the study. The consultant psychiatrist will be approached in order to make sure the patient have got capacity to consent for research. Written Informed Consent will then be obtained from both member of staff and patients by means of participant dated signature and dated signature of the person who presented and obtained the Informed Consent. The person who obtained the consent must be suitably qualified and experienced, and have been authorised to do so by the Chief/Principal Investigator. A copy of the signed Informed Consent will be given to the participant. The original signed form will be retained at the study site.

**6.3 Visits**

After consent is in place the researcher will meet the member of the patient’s regular care team. The researchers will not interview the patients themselves. By using the foxweb tool, they will then go through a list of questions on the iPad about information that is routinely collected about a particular patient (this will only take few minutes). The researcher will input the data as he/she asks the questions. This process should only take a maximum of 2 minutes per patient. This will be done for the same patients regularly on a regular (1-4 weekly) basis and will be how data is gathered. In total, it would take a maximum of 30 minutes to cover all the patients on a particular ward. At the end of follow up, we will download routinely collected information on critical incidents (violence towards staff, other patients and property, self harm, and absconding) from a Trust-based database called 'Safeguard'. We will then examine associations between risk factors and critical incidents, particularly those involving violence towards others.

## 6.4 Discontinuation/Withdrawal of Participants from Study

Patients as well as staff have the right to leave the study whenever they feel appropriate. They merely have to let the researcher know. If a patient does withdraw, there data that has already been gathered will still be kept for the purposes of the research.

## 6.5 Definition of End of Study

The Study will be completed when data on the last inpatients enrolled into the study has been collected.

#

# STATISTICS AND ANALYSIS

## Description of Statistical Methods

## After the data have been downloaded from safeguard about patients included in the study, associations will be examined using standard methods - univariate and multivariate regression to examine associations between individual risk factors and outcomes; measures of calibration and discrimination of the included risk factors (sensitivities, specificities, positive and negative predictive values, Harrel's C index) to test predictive accuracy of the tool for critical incidents.

# DATA MANAGEMENT

## Access to Data

Direct access will be granted to authorised representatives from the Sponsor or host institution for monitoring and/or audit of the study to ensure compliance with regulations.

## Data Recording and Record Keeping

Electronic data will be stored on University computers in a secured building, and will be firewall and password protected. After the study is finished the data will be stored in filing cabinets and password protected computers, in locked rooms at the Department of Psychiatry in Oxford.

# ETHICAL AND REGULATORY CONSIDERATIONS

## Declaration of Helsinki

The Investigator will ensure that this study is conducted in accordance with the principles of the Declaration of Helsinki.

## Guidelines for Good Clinical Practice

The Investigator will ensure that this study is conducted in accordance with relevant regulations and with Good Clinical Practice.

## Approvals

The protocol, informed consent form, participant information sheet and any proposed advertising material will be submitted to an appropriate Research Ethics Committee (REC), and host institution(s) for written approval.

The Investigator will submit and, where necessary, obtain approval from the above parties for all substantial amendments to the original approved documents.

## Reporting

The CI shall submit once a year throughout the study, or on request, an Annual Progress report to the REC Committee, host organisation and Sponsor. In addition, an End of Study notification and final report will be submitted to the same parties.

## Participant Confidentiality

The study staff will ensure that participants’ anonymity is maintained. The participants will be identified only by initials and a participants ID number on the CRF and any electronic database. The study will comply with the Data Protection Act which requires data to be anonymised as soon as it is practicable to do so. Electronic data will be stored on University computers in a secured building, and will be firewall and password protected.

# FINANCE AND INSURANCE

## Funding

Funding will be by the department of Psychiatry Oxford.

## Insurance

The study will be insured by Oxford University R & D department. The University of Oxford maintains Public Liability and Professional Liability insurance, which will operate in this respect.

# 11. Time line

Month 1-Asking consultant psychiatrist about capacity for consent for research. Gaining patients consent for study

Month 2-Data collection, regularly collecting information from patient regular care team using Foxweb instrument

Month 3- Data collection, regularly collecting information from patient regular care team using Foxweb instrument

Month 4- Data collection, regularly collecting information from patient regular care team using Foxweb instrument

Month 5-Download data from safeguard about patients included in study

Month 6-Analysis of data

**Appendix D**

Analysis Plan

**Feasibility, scalability and utility of a web-based violence risk monitoring tool in adult psychiatric inpatients (FoxWeb)**

**1. Study Summary**

**1.1 Design**

Prospective cohort study.

**1.2 Data**

Data collected were

- Demographic and clinical characteristics: age, sex, ward type, and ICD-10 psychiatric diagnosis;
- Historical information: previous conviction for interpersonal violence, history of receiving inpatient treatment under the Mental Health Act, substance use disorder, alcohol use disorder, high baseline anger, and a history of self-harm;
- Dynamic factors: non-adherence with therapy; non-adherence with medication; aggression (verbal or physical); discharge of tension and emotions; emergence/deterioration of paranoid/persecutory delusions; emergence/deterioration of hallucinations; increasing anger due to psychotic symptoms; emergence/increase in drug misuse; emergence/increase in alcohol misuse; increase in anxiety. Dynamic factors were administered at regular intervals and transferred to a web-based monitoring system called the Forensic Oxford Web (FoxWeb) tool to calculate a total dynamic score.
- Information on violent incidents: gathered from a routine incident-reporting system.

Data structure was repeated individual item and total dynamic scores for violence risk assessment for psychiatric patients clustered within wards (either forensic or general).

**1.3 Participants**

Consenting adult (18 years old and over) inpatients on (6 male and 2 female) forensic and (2 male and 2 female) general wards within the Oxford Health NHS Trust over three sites in two English counties.

**1.4 Study period**

The study period is between 1st March 2015 and 31st December 2016.

**1.5 Risk factors**

Risk factors were

- Calendar age, in decades
- Sex (1=female; 0=male)
- Ward type (1=forensic; 0=general)
- ICD-10 psychiatric diagnosis (1=psychosis; 0=other main categories)
- Total dynamic score calculated as the sum of the 10 individual dynamic factors each ranging 0-4, ignoring individual item non-response. This factor will be separately considered in three forms:
  - As a continuous variable, ranging 0-40.
  - As a dichotomous variable taking the value of 1, if Total dynamic score > 0; 0, otherwise.
  - As a dichotomous variable taking the value of 1, if Total dynamic score > 4; 0, otherwise.

**1.6 Primary and secondary outcomes**

The primary outcome is occurrence of violent/aggressive incident (physical or verbal), independently of the person being the instigator or not. This variable takes the values

- 1, if an incident occurred within 1 week from dynamic risk assessment and before next assessment for acute wards; or
- 1, if an incident occurred within 4 weeks from dynamic risk assessment and before next assessment for forensic wards; or
- 0, if no incident occurred or if an incident occurred but outside the above time frames,

The window following the dynamic risk assessment may vary between patients, depending on the number and frequency of assessments per patient. The primary outcome will be linked to the prior most recent dynamic risk assessment.

It is expected that, for some participants, more than one risk assessment is recorded at the same time (or close in time).  When there are repeated risk assessments within 24 hours from each other, we will use only the one with the latest time-stamp, thought to be the most likely to be reliable.

The secondary outcome is being an instigator (1=the patients was the instigator of the violent/aggressive incident; 0=otherwise).

**1.7 Study objectives**

*Primary objective*

The main objective is to assess the association between total dynamic score at risk assessment and the occurrence of violent incidents, adjusted for the remaining risk factors (see Section 1.5).

*Secondary objective*

To assess the association between total dynamic score at risk assessment and being the instigator of an incident.

*Exploratory objectives*

The following secondary will be performed wherever data availability allows it.

- To investigate the correlation between number of risk assessments and number of incidents.
- To explore the time elapsed from risk assessment to violent incident.
- To estimate the prevalence of incidents, overall, by type and by category.
- Any analysis of the 10 individual components of the total dynamic score in relation to violent incident occurrence will be treated as exploratory and used to guide future prospective work.
- To repeat the main analyses considering as incidents only those in which the patient in question was the instigator of the incident.

1. **Statistical Analysis**

**2.1 Descriptive analysis**

Summary statistics will be calculated for all variables in the dataset as follows: number and percentage, for categorical variables; mean (standard deviation) and median (interquartile range), for continuous variables.

**2.2 Primary analysis**

For the primary analysis, multilevel logistic regression models will be fitted with patient as the grouping variable, as follows.

Univariable analyses will be performed to evaluate the independent association of each risk factor (Section 1.5) and incident occurrence. Odds ratio (OR) estimates and corresponding 95% confidence intervals (95%CI) will be reported.

Multivariable analysis will be performed to assess the association of total dynamic score and incident occurrence, adjusted for age, sex, ward type and psychiatric diagnosis (as specified in Section 1.5). ORs and 95%CIs will be reported. No selection of variables will be performed.

**2.3 Secondary and exploratory analysis**

The primary analysis will be repeated independently replacing total dynamic score with each of its 10 individual components as well as with its dichotomised versions (i.e. total dynamic score >0 and total dynamic score >4).

We will select the model that best explains the association between total dynamic score (or each of it dichotomisations) by looking at their deviance and Akaike Information Criterion (AIC). For both parameters, the lower the better.

Time from risk assessment to incident occurrence will be explored by summarizing the elapsed time from prior dynamic risk assessment to each incident occurring before next assessment, considering type of ward and also ignoring type of ward.

Prevalence of incidents will be calculated as number and percentage for the overall sample, by sex, by decade of age, by main diagnosis categories, and by type of ward.

To investigate the association of total dynamic score and being the instigator of a violent incident, the primary analysis will be repeated replacing the outcome incident occurrence with being the instigator.

**2.4 Missing values**

Missing data will be summarized for all variables in the dataset. No missing values are expected in demographic and clinic characteristic. Due to the collection method, if non-response item for the individual elements of the total dynamic scores is small, it will be assumed missing completely at random. No imputation method will be implemented.

**2.5 Model performance**

The prediction performance of the fitted models will be assessed as follows.

Discrimination – the ability to differentiate patients with and without the outcome- will be assessed calculating the area under the ROC curve, considering both random and fix effects and also excluding random effects. The latter will give an idea of the performance of the model in practice, where the model would be evaluated at a particular risk assessment for a single individual.

Calibration – the ability to predict probabilities close to the observed ones - will be assessed plotting the observed versus predicted probabilities in deciles.

NB: We had planned to calculate sensitivity and specificity but no cut-point was pre-specified. It was decided to exclude this analysis to avoid data driven values.
